# Depletion of the *Nb* CORE receptor drastically improves agroinfiltration productivity in older *Nicotiana benthamiana* plants

**DOI:** 10.1111/pbi.14037

**Published:** 2023-03-14

**Authors:** Isobel Dodds, Changlong Chen, Pierre Buscaill, Renier A. L. van der Hoorn

**Affiliations:** ^1^ The Plant Chemetics Laboratory Department of Biology University of Oxford Oxford UK; ^2^ Beijing Academy of Agriculture and Forestry Sciences Beijing China; ^3^ Beijing Key Laboratory of Agricultural Genetic Resources and Biotechnology Institute of Biotechnology Beijing China

**Keywords:** *Agrobacterium tumefaciens*, agroinfiltration, *Nicotiana benthamiana*, cold shock protein, CORE receptor, transient GFP expression

## Abstract

*Nicotiana benthamiana* is increasingly used for transient gene expression to produce antibodies, vaccines, and other pharmaceutical proteins but transient gene expression is low in fully developed, 6–8‐week old plants. This low gene expression is thought to be caused by the perception of the cold shock protein (CSP) of *Agrobacterium tumefaciens*. The CSP receptor is contested because both *Nb*CSPR and *Nb*CORE have been claimed to perceive CSP. Here, we demonstrate that CSP perception is abolished in 6‐week‐old plants silenced for *Nb*CORE but not *Nb*CSPR. Importantly, older *Nb*CORE‐silenced plants support a highly increased level of GFP fluorescence and protein upon agroinfiltration. The drastic increase in transient protein production in *Nb*CORE‐depleted plants offers new opportunities for molecular farming, where older plants with larger biomass can now be used for efficient protein expression.

*Nicotiana benthamiana* is frequently used for transient gene expression (Bally *et al*., 2018). In addition to studies on subcellular localization, protein–protein interaction and enzymatic activities, transient gene expression is commercially used to produce antibodies, vaccines, and other pharmaceutical proteins (Sainsbury, 2020; Schillberg and Spiegel, 2022). Transient expression is achieved by infiltrating leaves or whole plants with disarmed *Agrobacterium tumefaciens* harbouring a binary vector that carries genes‐of‐interest on the transfer DNA (T‐DNA). *A. tumefaciens* transfers this T‐DNA into the plant cell, where it is expressed.

The success of transient gene expression decreases with the age of the *N. benthamiana* plants, despite having more biomass and large leaves that are easy to infiltrate (Lai and Chen, 2012; Saur *et al*., 2016). Best expression is achieved in 3–5 week‐old plants and poor expression in older, 6–8‐week‐old plants that start flowering. The poor gene expression is thought to be caused by the perception of cold shock protein (CSP) of *A. tumefaciens* (Saur *et al*., 2016). A 22 amino acid fragment of CSP called csp22 is sufficient to trigger immune responses including a burst of reactive oxygen species (ROS) (Felix and Boller, 2003; Saur *et al*., 2016). The csp22‐induced ROS burst is observed from leaf discs from old plants, but not from young plants (Saur *et al*., 2016), implicating that CSP recognition might indeed underpin the success of transient gene expression in older plants.

Two distinct receptors, the receptor‐like protein *Nb*CSPR (Saur *et al*., 2016) and the receptor‐like kinase *Nb*CORE (Wang *et al*., 2016), respectively, have been proposed to act as CSP receptors. Transcripts of both receptors, encoding proteins with only 29.9% amino acid identity, are detectable only in older *N. benthamiana* plants. *Nb*CSPR was reported to interact with csp22 and to be required for its perception because depletion of *Nb*CSPR by virus‐induced gene silencing (VIGS) suppressed the csp22‐induced ROS response. The silencing of *Nb*CSPR also resulted in higher transient expression in older plants after Agroinfiltration of a reporter gene. In disagreement with this report, Wang *et al*. (2016) could not confirm the role of *Nb*CSPR in csp22 perception. Rather, they report that CORE tomato (*Sl*CORE) forms the specific, high‐affinity receptor binding site that is required and sufficient for csp22 perception (Wang *et al*., 2016). They also found that its ortholog in *N. benthamiana*, *Nb*CORE, but not *Nb*CSPR, confers csp22 responsiveness when transformed into *Arabidopsis thaliana*, which is otherwise insensitive to csp22 (Wang *et al*., 2016).

Here, we depleted *Nb*CSPR and *Nb*CORE by VIGS to investigate if CSP perception hampers recombinant protein production in older plants. To silence *Nb*CSPR, we used the same 299 bp gene fragment of *Nb*CSPR used earlier (Saur *et al*., 2016; Tables S1 and S2), and cloned this into a vector expressing *RNA2* of tobacco rattle virus (TRV2gg). Similarly, a 300 bp fragment specific to *Nb*CORE was cloned into TRV2gg. Alignments of the used silencing fragments with the coding sequences of *Nb*CORE and *Nb*CSPR show that cross‐silencing is unlikely (Figure S1). TRV carrying a fragment of beta‐glucuronidase (*TRV::GUS*) was included as a negative control. 2‐week‐old *N. benthamiana* plants were infected with TRV carrying the silencing fragments. The *TRV::NbCSPR* and *TRV::NbCORE* plants have no developmental phenotypes compared to *TRV::GUS* plants (Figure 1a).

**Figure 1:**
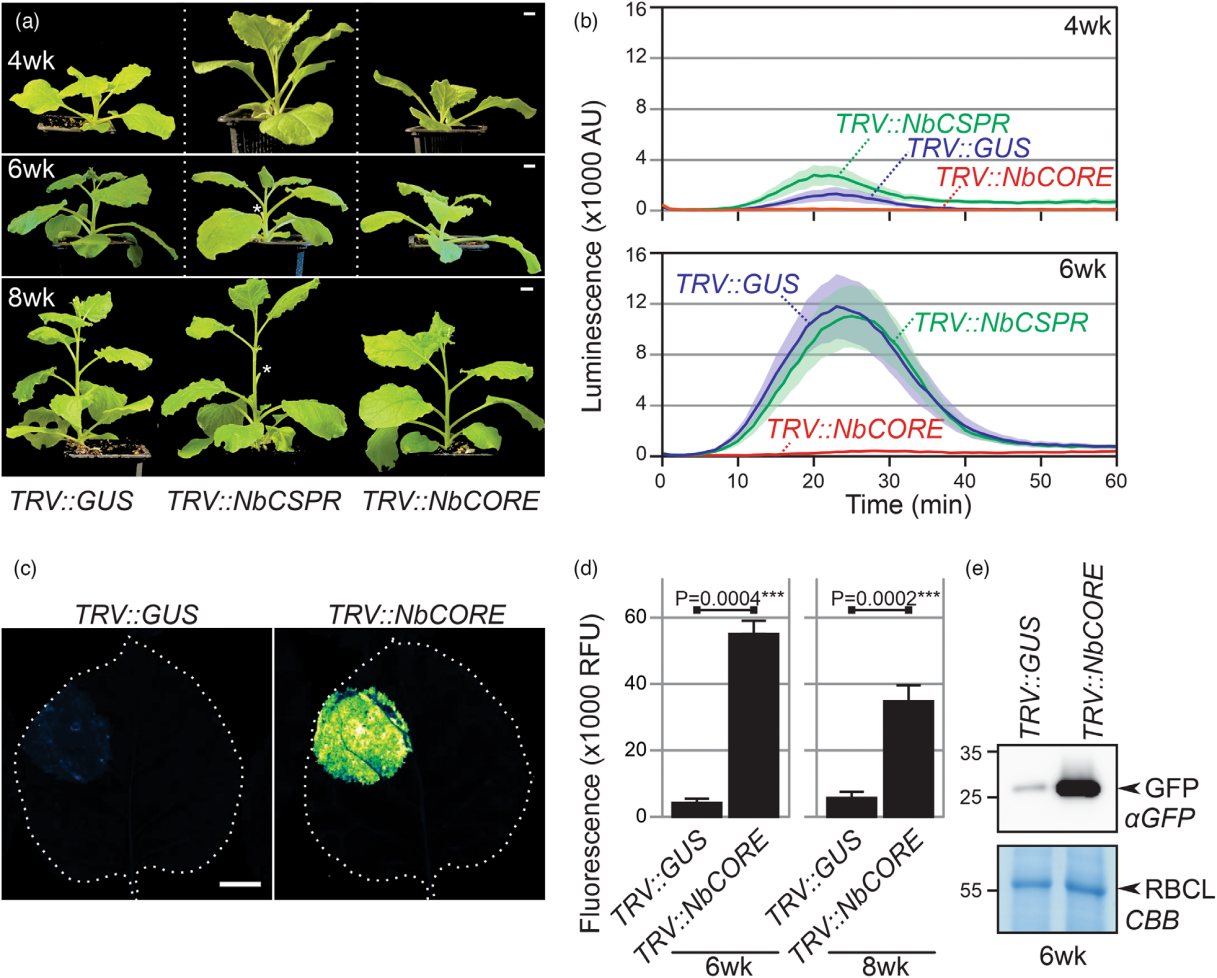
*Nb*CORE silencing removes csp22 responsiveness and increases transient protein production in older *N. benthamiana* plants. (a) *TRV::NbCSPR* and *TRV::NbCORE* plants have no additional developmental phenotype compared to *TRV::GUS* plants. Scale bars, 1 cm. *, removed sample leaves. (b) The csp22‐induced oxidative burst is absent from 6‐week‐old *TRV::NbCORE* plants but present in *TRV::GUS* and *TRV::NbCSPR* plants. Error shades represent the standard error of *n* = 6 leaf discs. (c) *Nb*CORE depletion causes bright GFP fluorescence upon agroinfiltration of 6‐week‐old plants. The image was taken 5 days agroinfiltration with 35S:eGFP. Scale bar, 1 cm. (d) Significant increase in GFP fluorescence upon *Nb*CORE depletion. GFP fluorescence was quantified from images of *n* = 4 biological replicates of 6‐week and 8‐week‐old VIGS plants agroinfiltrated with 35S:eGFP 5 days before fluorescence scanning. Fluorescence was quantified using ImageJ and normalized by leaf area. ****, *P* value = 0.0000084 (*t*‐test). (e) *TRV::NbCORE* plants accumulate much more GFP protein upon agroinfiltration than *TRV::GUS* plants. Total leaf proteins were extracted from VIGS plants, 5 days after agroinfiltration with *35S:eGFP*, and analysed by anti‐GFP western blot. CBB, Coomassie brilliant blue.

Leaf discs from 4‐week and 6‐week‐old VIGS plants were tested for a csp22‐induced oxidative burst. Importantly, the csp22‐induced ROS burst is absent from 6‐week‐old *TRV::NbCORE* plants and is present in *TRV::NbCSPR* plants, comparable to *TRV::GUS* control plants (Figure 1b and Figure S2). As reported before, younger, 4‐week‐old plants, only have very weak csp22‐induced responses that can vary per batch of plants (Figure 1b and Figure S2). These data demonstrate that *Nb*CORE is essential for the csp22‐induced oxidative burst in older *N. benthamiana* plants. Our data indicate that *Nb*CSPR is not necessary for csp22 perception, consistent with the findings of Wang *et al*. (2016) that *Nb*CSPR is not sufficient for csp22 perception.

To investigate to what level the depletion of *Nb*CORE promotes transient gene expression, we agroinfiltrated 6w‐old *TRV::GUS* and *TRV::NbCORE* plants with Agrobacterium delivering enhanced GFP driven by a strong 35S promoter (35S:eGFP, Kourelis *et al*., 2021), and scanned the agroinfiltrated leaves for fluorescence five days later. Bright GFP fluorescence was detected in *TRV::NbCORE* plants, whereas hardly any fluorescence was detected in *TRV::GUS* plants (Figure 1c), corresponding to a nearly eight‐fold increased GFP fluorescence (Figure 1d). A similar increased fluorescence was observed upon infiltrating 8‐week‐old plants (Figure 1d). Western blot analysis confirmed a drastically increased GFP protein level in *TRV::NbCORE* plants compared to the *TRV::GUS* control plants (Figure 1e).

Our data showing that *Nb*CORE is required for csp22‐induced oxidative burst is consistent with reports that *Nb*CORE binds csp22 with high affinity (Kd = 6 nM, Wang *et al*., 2016) and that transient expression of *Nb*CORE confers csp22‐responsiveness to leaves of young plants (Wei *et al*., 2018). The csp22‐induced ROS burst in *TRV::NbCSPR* plants was similar to the *TRV::GUS* control, which contradicts earlier work (Saur *et al*., 2016). Our data is, however, consistent with experiments that *Nb*CSPR is unable to confer csp22‐responsiveness (Wang *et al*., 2016). A more recent report also shows that *Nb*CSPR is identical to RE02, the receptor for VmE02, a conserved Cys‐rich protein secreted by diverse microbes (Nie *et al*., 2021).

Our results offer new opportunities in molecular farming, where older plants with larger biomass can now be used for efficient transient gene expression. A more durable depletion of *Nb*CORE can be achieved by genome editing, or by engineering *A. tumefaciens* strains to contain a CSP that is no longer recognized by *Nb*CORE. Both approaches will drastically improve transient protein production in older *N. benthamiana* plants, without the need for a licence to work with TRV to deplete *Nb*CORE by VIGS.

## Conflict of interest

The authors declare no conflict of interest.

## Author contributions

ID and CC performed experiments and analysed the data; ID, PB and RH designed experiments; ID and RH wrote the paper with help from all authors. All the authors read and approved the final manuscript.

## Supporting information


**Appendix S1.** Supplemental Methods, Figures and Tables.
