# Supplementary material for: Depletion of the Nb CORE receptor drastically improves agroinfiltration productivity in older Nicotiana benthamiana plants
Source: Plant Biotechnol J. 2023 Mar 14;21(6):1103–5. doi: 10.1111/pbi.14037 (PMC10214749; doi:10.1111/pbi.14037)
Supplement: Supplementary file 1 — Appendix S1. Supplemental Methods, Figures and Tables. [file PBI-21-1103-s001.docx]

Supplemental Materials

**Depletion of the *Nb*CORE receptor drastically improves agroinfiltration productivity in older *Nicotiana benthamiana* plants.**

Isobel Dodds, Changlong Chen, Pierre Buscaill, Renier A. L. van der Hoorn

**Supplemental Materials & Methods**

***Plant cultivation conditions –*** Wild-type *Nicotiana benthamiana* (LAB) seeds were sown into a 3:1 mix of soil (Sinclair Modular Seed Peat reduced propagation mix) with vermiculite (Sinclair brand Pro Medium) in 7x7 cm square pots and grown at high humidity under transparent plastic covers for 5 days. Seedlings were uncovered and grown first for one week in the greenhouse at 80-120 μmol/m^2^/s light and 21°C (night) and 22-23°C (day) in a 16 hour light regime. Plants were then agroinfiltrated with TRV vectors and grown in a growth chamber at 100 µmols/m^2^/sec light and 21°C and 50-60% relative humidity in a 16 hr light regime. Plants were watered three times per week such that no pots were standing in water overnight.

***Construction of plasmids -*** The fragments in **Table S1** were synthesized by Twist Biosciences and cloned into the golden-gate compatible vector TRV2gg (Duggan et al., 2016) in a BsaI reaction to produce expression plasmids. The fragment sequence published by (Saur et al., 2016) was used to silence *NbCSPR*. The fragment for silencing *NbCORE* was designed using the SolGenomics VIGS tool, SGN-VIGS (Fernandez-Pozo et al., 2015). Plasmids were transformed into *E. coli* DH10β for amplification, were purified and then transformed into *Agrobacterium tumefaciens* GV3101-pMP90. Transformants were selected on plates of lysogeny broth (LB) agar medium containing 25 μM rifampicin, 10 μM gentamycin and 50 μM kanamycin. A single colony of transformant was cultured in liquid LB containing the same antibiotics.

***Virus-induced gene silencing -*** Agrobacteria cultures were grown overnight at 28°C in LB medium containing 25 μM rifampicin, 10 μM gentamycin and 50μM kanamycin. The cultures were centrifuged at 3500 x *g* for 10 minutes at room temperature and then resuspended in infiltration buffer (10 mM MES, 10 mM MgCl_2_, 100 μM acetosyringone at pH 5.7). All cultures were diluted to OD_600_=0.5 and a culture carrying a plasmid encoding TRV1 was mixed at a 1:1 ratio with cultures carrying plasmids encoding TRV2 containing fragments to silence glucuronidase *GUS* (negative control), *NbCSPR, NbCORE* and phytoene desaturase *PDS* (positive control). *Nicotiana benthamiana* plants (LAB) were grown at 21°C under a 16/8 hour light/dark routine in a greenhouse. Both true leaves of 14-day-old plants were agroinfiltrated with the bacterial suspension using a 1 ml syringe without a needle. Three and five weeks later plants were assessed for silencing by checking for bleached leaves in *TRV::PDS* plants.

***ROS assays –*** The ROS burst assay was performed as described (Buscaill et al., 2019) with the difference that L-012 (Wako Chemical, Japan) was used instead of luminol and the diameter of leaf discs used here was 4 mm rather than 6 mm. Briefly, after incubation in water overnight, one leaf disc (4 mm diameter) was added to 100 µl solution containing 25 ng/µl L-012, 25 ng/µl Horse Radish Peroxidase (HRP) and 500 nM csp22. Chemiluminescence was measured immediately with the Infinite M200 plate reader (Tecan, Mannedorf, Switzerland) every minute for one hour. The used csp22 peptide is from *Pto*DC3000 (LNGKVKWFNNAKGYGFILEDGK) and was synthesized by Genscript.

***GFP expression and analysis –*** Agrobacterium GV3101(pMP90) carrying pJK-B2-022 encoding eGFP (Kourelis et al., 2021) were grown overnight at 28°C in LB medium containing 25 μM rifampicin, 10 μM gentamycin and 50 μM kanamycin. The cultures were centrifuged at 3500 x *g* for 10 minutes at 21 °C and then resuspended in infiltration buffer (10 mM MES, 10 mM MgCl_2_, 100 μM acetosyringone at pH 5.7) to a OD_600_=0.5. Expanded leaves of VIGS plants were agroinfiltrated using a needleless syringe. At 5 days post infiltration (dpi) leaves were scanned for fluorescence on a Amersham Typhoon 5 Biomolecular Imager (GE Healthcare Life Sciences, Little Chalfont, UK) using the Cy2 settings. Quantification of fluorescence was performed using ImageJ and normality was tested by a Shapiro-Wilk test and the probability value was calculated with a Well’s t-test. From the same leaves, 1 cm leaf discs were punched, flash-frozen in liquid nitrogen and ground using a pestle. The leaf tissue powder was mixed 3:1 with phosphate-buffered saline and centrifuged for 10 minutes at 13,000 x *g* at 4°C. The total soluble protein supernatant was mixed 1:3 in 4x gel loading buffer (200 mm Tris-HCl (pH 6.8), 400 mm DTT, 8% SDS, 0.4% bromophenol blue, 40% glycerol) and heated at 95°C for 5 minutes.

***Western blot analysis -*** Proteins were separated on a 12% w/v SDS-PAGE gel, transferred onto a polyvinylidene difluoride (PVDF) membrane using the TransBlot Turbo system (Bio-rad, Hercules, CA) and blocked for 1 hour at room temperature in 5% w/v skimmed milk in phosphate-buffered saline (PBS) with 0.01% v/v Tween-20. eGFP was detected using an α-GFP-HRP antibody (Abcam ab6663, 1/5000) also in 5% w/v skimmed milk in PBS with 0.01% v/v Tween-20. Chemiluminescent signals were detected using the SuperSignal™ West Femto Maximum Sensitivity Substrate (Thermo Fisher Scientific, Waltham, MA, USA). eGFP-antibody complex signals were captured using the ImageQuant LAS 4000 (GE Healthcare, Healthcare Life Sciences, Little Chalfont, UK). A corresponding 12% w/v SDS-PAGE gel was run simultaneously and incubated in InstantBlue® Coomassie Protein Stain (Abcam, Cambridge, UK) and then imaged using the Epson Perfection V600 Photo scanner (Epson, Nagano, Japan).

**References**

**Buscaill P, Chandrasekar B, Sanguankiattichai N, Kourelis J, Kaschani F, Thomas EL, Morimoto K, Kaiser M, Preston GM, Ichinose Y, van der Hoorn RAL.** (2019) Glycosidase and glycan polymorphism control hydrolytic release of immunogenic flagellin peptides. *Science* **364**:eaav0748.

**Duggan C, Tumlas Y, Bozkurt TO.** (2021) A golden-gate compatible TRV2 virus induced gene silencing (VIGS) vector. Zenodo 10.5281/zenodo.5666891.

**Fernandez-Pozo N, Rosli HG, Martin GM, Mueller LA.** (2015) The SGN VIGS tool: user0feindly software to design virus-induced gene silencing (VIGS) constructs for functional genomics. *Mol. Plant* **8**:486-488.

**Kourelis J, Marchal C, Kamoun S.** (2021) NLR immune receptor-nanobody fusions confer plant disease resistance. bioRxiv 2021.10.24.465418

**Saur IM, Kadota Y, Sklenar J, Holton NJ, Smakowska E, Belkhadir Y, Zipfel C, Rathjen JP.** (2016) *Nb*CSPR underlies age-dependent immune responses to bacterial cold shock protein in *Nicotiana benthamiana*. *Proc Natl Acad Sci USA.* **113**:3389-94.


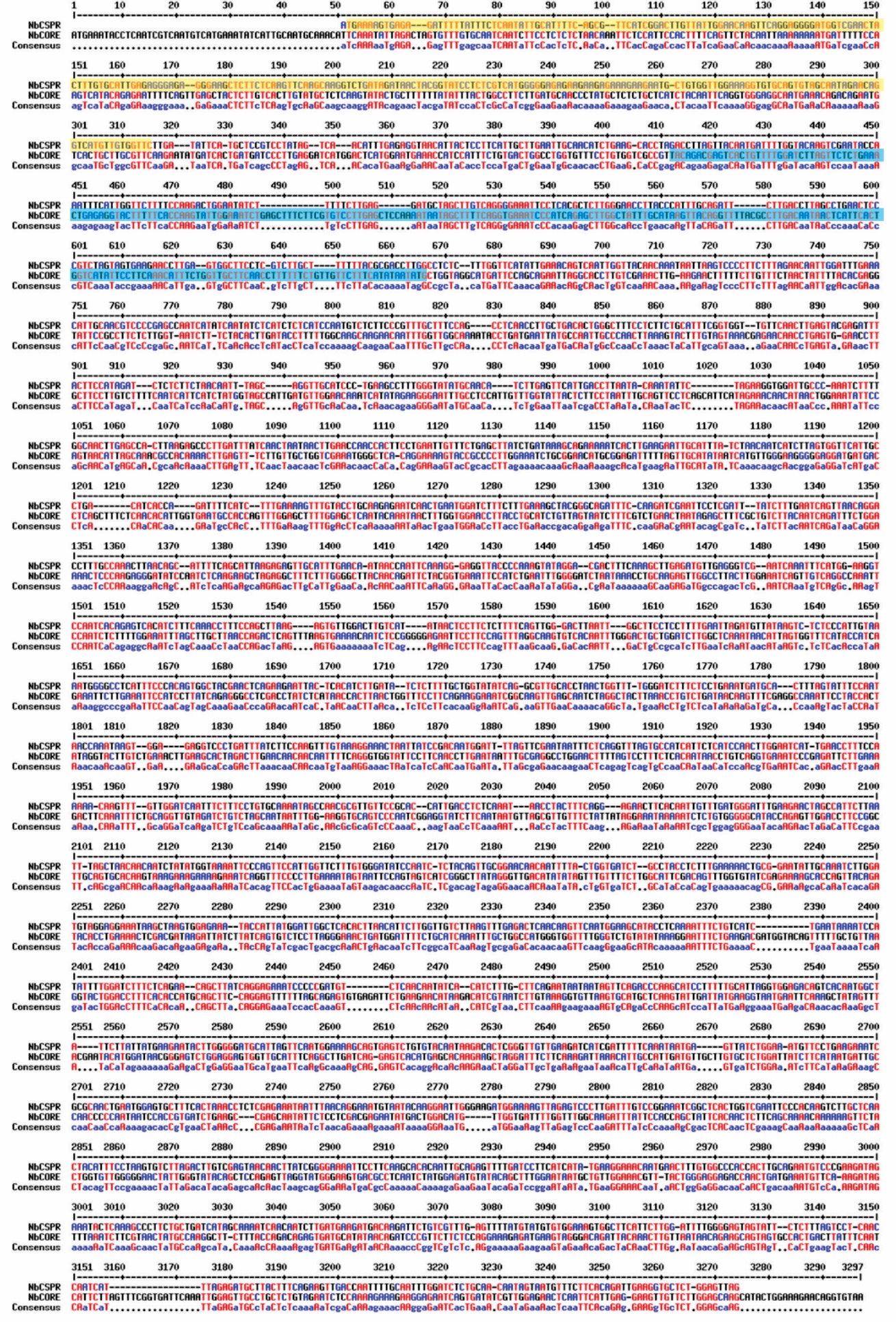


**Figure S1** Nucleotide alignment between *Nb*CSPR and *Nb*CORE

Nucleotide sequences of the open reading frames of *Nb*CSPR (NbD023129) and *Nb*CORE (NbD017538) were aligned using MultAlin. Fragments used for silencing are highlighted.

**
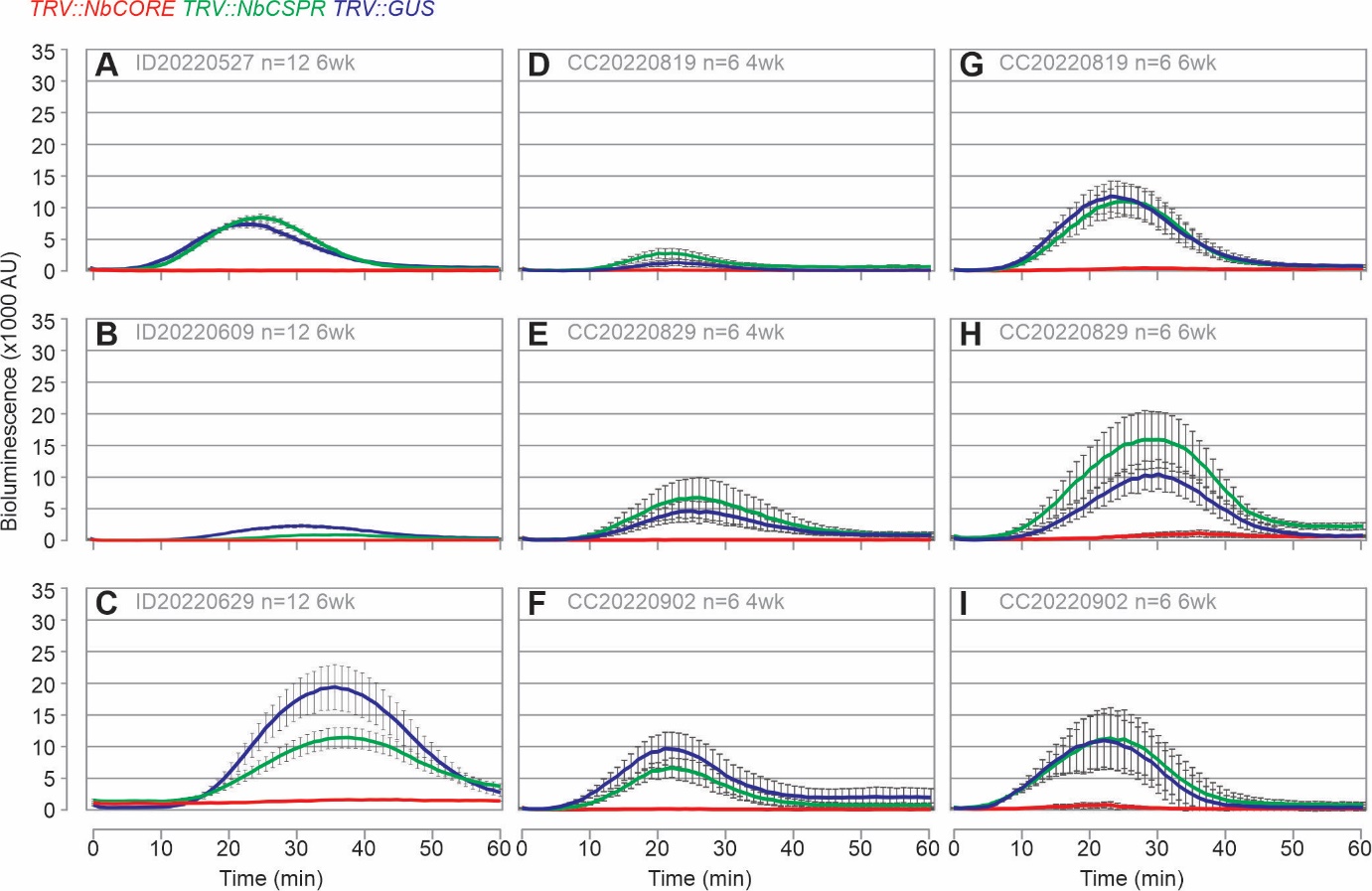
**

**Figure S2** Replicate ROS experiments.

Two week (2wk) old plants were agroinfiltrated with a 1:1 mixture of agrobacterium cultures delivering TRV1 and TRV2, respectively. TRV2 contains fragments GUS (blue), *Nb*CSPR (green) or *Nb*CORE (red). Leaf disks were taken from 4wk (D-F) or 6wk (A-C, G-I) old plants, incubated overnight in water and transferred to a 96-well plate. ROS assay solution containing L-012, HRP and 500 nM csp22 peptide of *Pto*DC3000 was added and the luminescence was measured every minute for 60 minutes immediately. Error bars represent standard error of n=12 (A-C) and n=6 (D-I) replicate leaf disks from 3-4 plants. Datasets D and G are also shown as main figure.

**Table S1** Used oligonucleotides

| >*Nb*CSPR fragment for VIGS  TGAAAAGTGAGAGATTTTTATTTCTCAATATTGCATTTTCAGCGTTCATCGGACTTGTTATTGGAACAAGTTCAGGAGGGGATGGTCGAACTACTTTGTGCATTGAGAGGGAGAGGGAAGCTCTTCTCAAGTTCAAGCAAGGTCTGATAGATAACTACGGTATCCTCTCGTCATGGGGGAGAGAAGAAGAGAAAGAAGAATGCTGTGGTTGGAAAGGTGTGCAGTGTAGCAATAGAACAGGTCATGTTGTGGTTCTTGATATTCATGCTCCGTCCTATAGTCAACATTTGAGAGGTAAC |
| --- |
| >*Nb*CORE fragment for VIGS  TACAGACGAGTCACTGTTTTGGATCTTAGTTCTCTGAAACTGAGAGGTACTTTTTCACCAAGTATTGGAAATCTGAGCTTTCTTCGTGTCCTTGAGCTCCAAAATAATAGCTTTTCAGGTGAAATCCCATCAGAGCTTGGCTATTTGCATAAGTTACAGGTTTTACGCCTTGACAATAACTCATTCACTGGTCATATTCCTTCAAACATTTCTGGTTGCTTCAACCTTTTTTCTGTTGTTCTTTCATATAATATGCTGGTAGGCATGATTCCAGCAGAATTAGGCACCTTGTCGAAACTT |
| >GUS fragment for VIGS  TAAAGAGCTGATAGCGCGTGACAAAAACCACCCAAGCGTGGTGATGTGGAGTATTGCCAACGAACCGGATACCCGTCCGCAAGGTGCACGGGAATATTTCGCGCCACTGGCGGAAGCAACGCGTAAACTCGACCCGACGCGTCCGATCACCTGCGTCAATGTAATGTTCTGCGACGCTCACACCGATACCATCAGCGATCTCTTTGATGTGCTGTGCCTGAACCGTTATTACGGATGGTATGTCCAAAGCGGCGATTTGGAAACGGCAGAGAAGGTACTGGAAAAAGAACTTCTGGCCTGGCAGGAGAAACTGCATCAGCCGATTATCATCACCGAATACGGCGTGGATACGTTAGCCGGGCTGCACTCAATGTACACCGACA |

**Table S2** Constructs used

| **Name** | **Description** | **Reference** |
| --- | --- | --- |
| pID010 | *TRV2gg::NbCSPR* | This work |
| pID024 | *TRV2gg::NbCORE* | This work |
| TRV::GUS | *TRV2gg::GUS* | Duggan et al., 2021 |
| TRV1 | *RNA1 of TRV* | Liu et al., 2002 |
| pJK-B2-022 | *35S::eGFP* | Kourelis et al., 2021 |
| TRV2::PDS | *TRV2::PDS* | Liu et al., 2002 |
